# Effects on neurocognition of theta burst transcranial magnetic stimulation in patients with resistant to treatment depression

**DOI:** 10.1192/j.eurpsy.2025.345

**Published:** 2025-08-26

**Authors:** Y. Cañada Perez, S. Albert, R. Roca, A. Sabater, P. Benavent, J. Ribes, P. Sierra

**Affiliations:** 1 Psychiatry and Psychology, La Fe University and Polytechnic Hospital; 2 Psychiatry, Dr Peset University Hospital, Valencia; 3 Psychology, Lluis Alcanyis Hospital, Xativa (Valencia); 4 Medicine, University of Valencia, Valencia, Spain

## Abstract

**Introduction:**

Cognitive dysfunction has been identified as a key mediator of functional impairment in major depression and a contributing factor to antidepressant resistance. Theta burst stimulation (TBS) is a novel form of transcranial magnetic stimulation (TMS) that has shown greater efficacy and efficiency than conventional TMS in the treatment of treatment-resistant depression (TRD). However, its effects on cognitive symptoms in depression remain largely unstudied.

**Objectives:**

The objective of this study is to evaluate the impact of TBS on neurocognition in unipolar and bipolar TRD patients treated at a public hospital. Additionally, clinical, demographic, and treatment predictors of cognitive change were explored.

**Methods:**

This is a follow-up study of n=64 patients with TRD (unipolar=48, bipolar=16) who received daily adjunctive TBS for 6 weeks. Cognitive performance was assessed before and after TBS using different versions of the Screening for Cognitive Impairment (SCIP-S), which measures immediate verbal learning, working memory, verbal fluency, delayed verbal learning, and processing speed. Cognitive performance in each domain was compared using paired t-tests. Global cognitive change was assessed by quantifying pathological domains at baseline and at the end of TBS. Differences in neurocognition between clinical responders (50% reduction on the HDRS) and non-responders to TBS were compared using ANOVA models. Additionally, possible predictors of cognitive change in each domain were explored using correlation analyses and multiple linear regressions, which included factors such as age, diagnosis, number of TBS sessions, treatment type, TBS modality (left unilateral vs. bilateral), clinical response, and baseline severity.

**Results:**

Patients treated with TBS achieved significant clinical response (68%) and cognitive improvement in the domains of immediate verbal learning, working memory, verbal fluency, and processing speed (mean differences of 2.16, 2.31, 1.30, and 1.05, respectively). Neurocognitive improvement was independent of clinical response. The percentage of improvement on the HDRS was only associated with improvement in the verbal fluency domain (p = .007). Left unilateral TBS and bipolar diagnosis predicted better global cognitive improvement in SCIP in regression models (p = .042, p = .037, respectively).

**Conclusions:**

The results support the utility of TBS in treating cognitive dysfunction associated with TRD. Further larger studies are needed to clarify clinical and treatment predictors of cognitive improvement.

**Disclosure of Interest:**

None Declared

